# Implementation of Virtual Focus Groups as an Effective Strategy in Qualitative Research to Engage With Undersupported Communities: Protocol for Virtual Focus Groups

**DOI:** 10.2196/77937

**Published:** 2025-12-08

**Authors:** Axel Ramos-Lucca, Jorge Luis Motta-Pagán, Karla Vélez-Mojica, Jacob Matos-Castro, Dorimar Rodríguez-Torruella, David Andres Vélez-Maldonado, Fernando Jose Rosario-Maldonado, Jeannie Aguirre-Hernández, Luisa Morales-Torres, Eida Castro-Figueroa, Elizabeth Rivera-Mateo, Melissa Marzán-Rodríguez, Julio Jiménez-Chávez

**Affiliations:** 1 School of Behavioral and Brain Sciences, Ponce Health Sciences University, 388 Zona Industrial Reparada 2, Ponce, 00716, Puerto Rico, 787-840-2575 ext 5591; 2 Ponce Research Institute - RCMI, Ponce Health Sciences University, Ponce, Puerto Rico; 3 Public Health Department, Ponce Health Sciences University, 388 Zona Industrial Reparada 2, Ponce, 00716, Puerto Rico; 4 School of Medicine, Ponce Health Sciences University, Ponce, Puerto Rico; 5 Department of Academic Affairs, Ponce Health Sciences University, Ponce, Puerto Rico

**Keywords:** virtual focus groups, qualitative research, protocols, undersupported communities, community engagement

## Abstract

**Background:**

Qualitative research offers a valuable lens for understanding human experiences, behaviors, and social contexts, drawing upon communication, social interaction, and sociological perspectives. Focus groups are a key method within qualitative research for exploring these complex topics. While traditional focus groups offer valuable insights into group dynamics and shared perspectives, they can be limited by logistical challenges, such as geographic constraints and participant availability. To mitigate these issues, virtual modalities have emerged as a viable alternative, offering greater flexibility and accessibility for diverse populations. However, they also highlight persistent challenges, such as managing group dynamics in online settings and ensuring participant engagement and privacy concerns. Our protocol considers these issues and implements strategies, such as cofacilitators, more engaged research assistants, and the use of important security measures (participant name obfuscation, sharing links only on the day of the session, and password protection) as a way to overcome said issues.

**Objective:**

Expanding the methods for data collection in qualitative research is essential for advancing health differences research in hard-to-reach populations and public health emergencies. In this paper, we aim to describe our experience when implementing an adapted protocol for conducting virtual focus groups, the barriers encountered, and how we obtained a participation rate of 86% (63/73) compared to 55% (40/73) when done in person.

**Methods:**

To achieve this, we adapted and implemented a focus group protocol that approaches undersupported populations (social vulnerability index ≥0.45) who face challenges participating in traditional focus groups and addresses reported barriers in scientific literature using virtual focus groups. Our protocol was implemented in an exploratory qualitative study conducted from 2020 to 2021 to understand the community’s health needs in southern Puerto Rico. This protocol ensures participant and research team interactions in circumstances that require it, including pandemics, geographically displaced populations, and patients who are bedridden. After evaluating the practical and ethical considerations, our research team established this new protocol for collecting qualitative data in a virtual focus group environment and promoting the inclusion of groups experiencing health differences.

**Results:**

As a result, we collected quality data and found added benefits. We obtained a participation rate of 86% (63/73) compared to 55% (40/73) when done in person. Our findings also reveal further benefits that could impact groups that generally do not have the chance to participate in focus groups.

**Conclusions:**

This approach to focus groups could aid researchers who wish to study these hard-to-reach participants and support qualitative health differences research.

## Introduction

### Background

Qualitative research offers a valuable lens for understanding human experiences, behaviors, and social contexts, drawing upon communication, social interaction, and sociological perspectives [1,2] . Focus groups are a key method within qualitative research for exploring these complex topics. One of the best approaches in qualitative research to understanding these specific behaviors and perspectives is the use of focus groups, where a group of participants (generally 6‐12) are interviewed simultaneously, with a moderator who guides the process and encourages interactions and discussion among participants [3,4]. While traditional focus groups offer valuable insights into group dynamics and shared perspectives, they can be limited by logistical challenges, such as geographic constraints and participant availability [5]. To mitigate these issues, virtual modalities have emerged as a viable alternative, offering greater flexibility and accessibility for diverse populations [6]. However, they also highlight persistent challenges, such as managing group dynamics in online settings and ensuring participant engagement [7,8]. Our protocol takes these issues into consideration and implements strategies, such as cofacilitators and more engaged research assistants to overcome said issues.

According to Creswell and Creswell [2], qualitative methods require a mindful recruitment procedure to appropriately select participants to ensure study questions and objectives are answered and achieved. Relating to individuals who participate in a qualitative study, four main aspects have been identified: (1) the setting in which the study is taking place, (2) the individuals being interviewed, (3) the actions of the participants, and (4) the development of the events that are taking place [2]. These 4 aspects ensure that the focus group can run optimally and that quality data can be obtained to ensure an understanding of the phenomenon being studied.

### COVID-19 Pandemic and Virtual Modalities

The COVID-19 pandemic further underscored the need for adaptive research methods, leading to the accelerated adoption of virtual focus groups to continue essential studies while prioritizing participant safety [9]. Similar to studies conducted during this period [10-13], our study used virtual focus groups to examine health needs with a hard-to-reach population. This modality helped protect participants and research teams by preventing any potential health risks related to COVID-19 while also complying with national governmental restrictions. The shift to a virtual modality also raised ethical concerns, such as ensuring informed consent and protecting participant privacy in online settings [14], all of which our team was conscious of and ensured participant protection.

One such study that demonstrates the successful implementation of these strategies was that of Crocker and Stout [10], who studied the feasibility of transitioning an at-home in-person visit asthma program into a virtual format. Through their efforts, they were able to identify benefits, such as convenience for the participants and safety, as virtual visits were considered safer. Challenges included the need for more effective assessment strategies, potential engagement problems, and digital literacy. The researchers recommended strategies, such as rapport building, technical support, and the possibility of hybrid models. Such challenges were relevant to our study as well, and we share our solutions, which included more involved communication with participants before and during the focus groups.

Building on Matos-Castro et al [15] methodology, we created a refined virtual focus guide based on our experiences and identified more benefits that strengthen the validity of the results. We proposed this method not as a substitute for standard focus groups but as an alternative approach. Therefore, researchers can apply this method to different circumstances that could warrant using remote focus groups for emergencies and study populations that could benefit from this modality.

### Role of the Internet in Conducting Research

Since the emergence of the internet, its role in research has been recognized as a potential tool for gathering information from users across different locations [16]. Internet usage has continued to grow and become intricately intertwined with people’s lives; therefore, it is not surprising that various studies have been conducted to assess how this can be used in focus groups [17-19]. By leveraging technological advancements, there is an opportunity to widen the boundaries of qualitative research, including focus groups [5]. With this technology, it is possible to address many problems presented by traditional focus groups.

Early studies dealing with virtual focus groups predominantly adopted an asynchronous approach using email, chat rooms, and websites [17,19,20]. The qualitative data obtained in these studies were like those of traditional focus groups. On many occasions, participants appreciated the convenience and accessibility of expressing their thoughts at their own pace when they felt ready.

Another benefit was that patients experiencing chronic health conditions and mobility issues could use these platforms as online peer support groups [17].

However, these previous methods had several limitations. As mentioned before, focus groups were used because of the interactions between the participants. Other factors can also serve as valuable data on specific topics, such as tone of voice, body mannerisms, and facial expressions, which can be lost in a text-based focus group. Although this does not necessarily decrease the data quality, it should not be overlooked. One alternative that has been studied and used, with similarities to traditional focus groups and permits convenience and accessibility to participants, is videoconference-style focus groups. With these stated disadvantages in the loss of nonverbal cues, it should be noted that recent advancements in videoconferencing tools have enabled real-time virtual “face-to-face” conversations that can closely mimic in-person interactions.

Nonverbal cues offer valuable information that can be used for qualitative data reporting, including facial expressions, gestures, posture, and tone, which allow for clarification of intent and facilitate rapport building [21]. In video-based modalities, some cues remain but are diminished by factors, such as the camera’s position, video quality, and participants’ choices not to turn on their camera or to turn it off during a session [8]. In audio-only groups (synchronous) or text-based groups (asynchronous or synchronous), such cues are reduced or completely removed. This can pose a problem for interpreting group reactions and interactions. Thus, although videoconferencing serves as a means to maintain some of these cues and represents an improvement over early text-based methods, limitations remain. Researchers should remain attentive to these differences and consider them when implementing this type of protocol, as some research contexts may necessitate the use of these cues.

Videoconference-style focus groups try to recreate the face-to-face focus group experience by using video software in which the moderator and participants have cameras and audio devices. Before the COVID-19 pandemic, literature describing this area was relatively scarce and had not been explored thoroughly, as stated in a comprehensive review of virtual focus groups by Rupert et al [6]. In this review, chat and video focus groups were compared with in-person focus groups, and costs were found to be similar. In virtual focus groups, preparation time was decreased due to less travel and allowed recruitment of harder-to-reach populations. They observed increased miscellaneous costs (participant webcams, services, etc), and a higher participant attrition.

Despite these hurdles, virtual focus groups offer a unique opportunity by reducing logistical constraints. In response to these hurdles, and using our experience, we found that the associated costs were not as high as predicted and proved cost-effective. This could be attributed to the increased use of videoconferencing software due to the pandemic to the point of a reported “abnormal consumer interest,” as stated by Tudor [22], while also considering that US citizens have reported an increase in internet use [23]. With better access to the internet, it is more probable that participants will have videoconference-ready devices.

In Puerto Rico, where this study took place, statistics indicate an overall internet penetration of 87.3%, increasing the feasibility of virtual engagement. Due to this high access to the internet, this served as a critical enabler for successful protocol implementation in the context of our study [24]. Furthermore, in our experience with the participants and researchers, the virtual environment ensured cost savings in areas such as travel and preparation of venues for the groups, offsetting other costs associated with virtual modalities.

By implementing strategies, such as providing tailored technical support to undersupported communities, using cofacilitation techniques to enhance participant engagement, and engaging community health workers to promote recruitment, we offer a protocol for enhancing accessibility and rigor in a virtual focus group research context.

## Methods

### Phase 1: Ethical Approval and COVID-19 Protocol

The study was first approved by the Ponce Health Sciences University (PHSU) Institutional Review Board (IRB) on April 14, 2021, and last approved on August 25, 2023. Due to the COVID-19 pandemic, an addendum was made in which we proposed moving our protocol to a virtual nature to be able to continue research activities. To obtain this approval, the team identified which platform would conform to privacy standards. When implementing the study, Zoom (Zoom Communications Inc) software contained the features necessary for addressing privacy concerns. Among these were (1) the encrypted nature of videoconferencing, (2) the ability to have closed rooms that could only be accessed via passwords provided by the research team, (3) moderation tools that would allow for videoconference control, and the capacity to create breakout rooms in case a participant requires support, aided by one of the facilitators. Only those who had confirmed participation would be given the password to enter. The team made sure that all video and audio recordings were done through the computer to restrict access to the research team. After these modifications, we were able to run the virtual focus groups. The research team handled all identifiable data (eg, contact numbers and names), and participants remained unknown to any other participants. During conversations, participants were assigned numbers and encouraged to use them during discussions to avoid saying their names.

### Phase 2: Community Engagement and Team Formation

As part of the PHSU-Research Center for Minority Institutions (RCMI), the Community Engagement Core (CEC) focuses on health differences associated with chronic health conditions among disadvantaged populations in the southern region of Puerto Rico. The project follows a Community-Based Participatory (CBPR) approach as a model to address community health needs and differences [25]. CEC obtains helpful information and opinions from communities in disadvantaged positions, which will be referred to as undersupported populations. In the context of this study, the term undersupported populations refers to communities whose social vulnerability index (SVI) had a score of 0.45 or more. The CDC (Centers for Disease Control and Prevention)/ATSDR (Agency for Toxic Substances and Disease Registry) SVI is a place-based index designed to identify socially vulnerable areas based on factors, such as poverty, unemployment, age, race, and infrastructure, among others [26]. The score of 0.45 was chosen, as it falls within half or more of the most vulnerable population, with SVI typically being separated into quintiles, with 0.4‐1 falling into the moderate to greatest vulnerability groups [27]. The CEC team is comprised of community members and academic researchers in an active partnership in which all parties weigh in on all processes. Community members are assembled into 2 fully community-led groups, including the Community-Trained Workforce (CTW) and the Community Scientific Advisory Committee (CSAC). Both CTW and CSAC are groups of local community leaders who have worked closely with CEC. These members have been recruited in the past and have completed training in core areas, such as research ethics, data collection, and community investigations. These team members serve as liaisons between the research and the community via disseminating study information and ensuring research materials are culturally appropriate and relevant to the communities. All parties had to agree on every phase before any investigation or intervention was completed.

### Phase 3: Study Design and Participant Recruitment

An exploratory qualitative design was implemented using focus groups [2]. Online focus group participants were selected from communities in the following southern municipalities of Puerto Rico, including Peñuelas, Juana Díaz, Santa Isabel, Guayanilla, Guánica, and Coamo. Participants were (1) required to be 21 years or older, (2) belonged to the particular communities that were under study, and (3) provided informed consent. Exclusion criteria included participants with impairments that would not permit informed consent, as discussed by Matos-Castro et al [15].

As part of creating the focus group discussion questions, including logistics and locations, the CEC team (academic researchers, CTW, and CSAC) collaboratively developed a relevant, comprehensible, culturally competent, and sensitive process. For this purpose, a series of meetings was held between all groups in which feedback and revisions were made to the process.

A convenience sampling method was used for recruitment. To achieve a broad range of health status experiences within the communities, various strategies were used to disseminate study information, including printed materials, social media posts, and community partners. This approach enabled us to obtain varied perspectives from members of the communities in our exploratory study.

### Phase 4: Research Team Composition and Analytical Reflexivity

The research team was composed of academic researchers with expertise in focus group dynamics, psychology, and community engagement, as well as trained community partners from the CTW and CSAC. Team participation was divided by phases in which some members of the research team, in tandem with the CTW and CSAC, oversaw the identification of potential communities and participant recruitment to ensure culturally relevant sites and community trust [28]. Moderators and research assistants participated only in the data collection phase. They ran and supported the virtual focus group sessions, ensuring they occurred effectively. Notably, research assistants were the only ones to have contacted participants before the sessions. Moderators and cofacilitators began the sessions without prior knowledge of which participants were in these groups.

Transcription was handled by research members who had not participated in the groups. Initial coding was conducted by 2 members who participated in the focus groups, and then, to ensure reliability, 3 research members who did not participate in the focus groups coded the material separately at a subsequent time using the same process. Disagreements were discussed and resolved via team discussions and the use of an expanded codebook definition [29]. Additionally, the ensuing results were discussed with CTW and CSAC in follow-up meetings to ensure an adequate and contextualized understanding.

### Phase 5: Virtual Focus Groups Procedure and Data Collection

#### Virtual Focus Group Process

**Figure 1.**
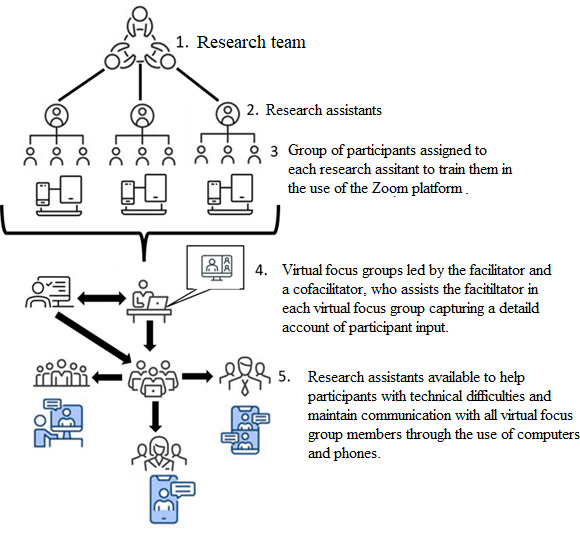
Workflow for the adapted virtual focus group protocol showing delegation of participant support to assistants and cofacilitation.

Figure 1 illustrates workflow implemented for the adapted virtual focus group protocol. The research team delegates assistants to participant groups who provide technical instruction and support to ensure comfort with Zoom prior to sessions, while a cofacilitator ensures smooth flow and research assistants address technical difficulties, sustain communications, and provide emotional support.

#### Research Team

##### Recruitment Strategies

Potential participants were invited to participate in the virtual focus groups using the strategies mentioned below.

##### Community Partners Networking

Members of the CTW leveraged existing community networks with other community leaders and associations to identify and reach potential areas of recruitment. This included phone calls with leaders, distribution of materials, WhatsApp messages in group chats, and social media sharing, all following COVID-19 safety protocols.

##### Social Media Platforms

Posts or virtual flyers were used to invite participants. These posts were shared through different platforms and pages (eg, Facebook [Meta Platforms, Inc], WhatsApp [Meta Platforms, Inc], and online community groups) frequently visited by target communities.

##### Printed Materials

Flyers and brochures were disseminated, following all safety protocols for COVID-19. The disseminated material contained contact information for the research team, which could be used to connect with potential participants and debrief them on the study’s essential and relevant information. After participants were debriefed and provided their informed consent, they were given an online form in which they would be able to sign digitally and receive a copy of the informed consent to their emails. If participants had no experience filling out online forms, the research team would guide them. Once all participants had been confirmed, the research team called them to obtain their availability and asked them to inform what time was most convenient for their participation. The research team cross-referenced the desired times and created a schedule accommodating participants.

### Research Assistant Training

Members of the research team were trained to fulfill the duties of notetakers in a focus group. Additionally, members were trained to be familiarized with the videoconference software and its functionalities while also making sure to understand the layout. In this way, they could monitor participants in both video and chat formats if needed. The members, including moderator and cofacilitator, were all prepared to offer psychological first aid and participant support to ensure both technological and emotional well-being.

### Research Assistant Follow-Up and Helping Participants

Participants were equitably assigned to trained researcher assistants, who instructed them to use the videoconferencing software (eg, Zoom) and the platform. Research assistants asked participants if they had questions about accessing or using the platform and provided recommendations for best practices for virtual focus groups, such as maintaining video cameras during the session, being in a private and quiet place, and communicating any troubles regarding connection.

Research assistants asked participants if they had the required hardware (eg, desktop, laptop, tablet, or smartphone) for virtual focus groups and if they had a stable internet connection. If participants answered no to any of these, the research team could loan the equipment necessary for these virtual focus groups for the duration of the groups. This equipment would only be lent out if necessary and returned to the research team after the study.

Before carrying out focus groups, research assistants called participants again to confirm whether they were available for the focus group session. Participants were given a password-protected link to Zoom videoconference. This platform was chosen because it uses encryption for communications and has protocols for the protection of confidential information [30]. Additionally, the research team implemented other strategies to ensure privacy, such as participants being assigned numbers before entering the chat and being recommended to use these numbers instead of names to ensure anonymity. Participants were encouraged to set apart a private physical space for privacy during sessions.

### Virtual Focus Group Sessions

During the focus groups, a cofacilitator was present to assist the main facilitator, who was running the virtual focus groups. The cofacilitator was ready to step in in case of technical difficulties for the moderator to keep participation active. If not, they only provided technical support.

Each session began with a moderator-led “ice-breaker” activity designed to commence dialogue and set a relaxed and inclusive tone, always emphasizing that all participation at any level is appreciated and that there were no right or wrong answers, promoting open sharing among participants and helping with any apprehension to participation.

### Support From Research Assistants During Focus Groups

Research assistants were on standby for any technical difficulties. If a participant had connection issues, research assistants contacted them through chat or phone to maintain their participation, help with troubleshooting, and transmit any messages typed in the chat.

### Support From the Research Team in Cases of Mental Health Distress or Crises

All personnel present in this phase were trained in psychological sciences and in managing potential crises to provide psychosocial support. Although no incidents occurred during these sessions, the team was equipped with a protocol for cases in which a participant was to confront a situation. In such cases, the research team could intervene, and if needed, move participants to a breakout room if they desired. If this were to occur, mental health aid would be provided, and the patient would be referred to a local wellness center associated with the institution for follow-up care. Additionally, all personnel involved in these groups had significant numbers for local services and hotlines. Forms were created to report these situations, detailing the incident and the steps taken.

### Considerations

The approach of using numbers as identifiers stems from earlier focus groups done in our research, in which, when given tags with numbers, participants would begin to use these as identifiers without instructions. In practice, participants in the virtual focus groups rarely used these, often using natural conversational references, such as “I agree with what she said.” This pattern of rapport might be due to local context and social norm; therefore, in other settings, pseudonyms or self-selected identifiers might be considered.

It is also important to note that the moderator was responsible for running focus groups and had the same responsibilities as that of a traditional moderator. Co-facilitators and Research Assistants provided technical support and note-taking during sessions, refraining from intervention except to communicate missed text or offer emotional support if needed. Any follow-up to dropouts was done to ensure that this was done willingly and not due to technical problems.

### Phase 6: Data Analysis

The focus group sessions were audio- and video-recorded using the Zoom platform. All audio data were transcribed manually via text editor software by members who did not participate in the focus groups and analyzed using the qualitative data analysis software Atlas.ti version 8 (ATLAS.ti Scientific Software Development GmbH). Content analysis was conducted through the lens of the Health Belief Model’s (HBM) theoretical approach to guide the focus groups’ domains and main topics, as described by Matos-Castro et al [15]. This approach has been used for past focus groups [31,32] and was developed using this model. Guided by the HBM, main domains and topics were identified and classified. Also, emergent topics not included in the HBM were included through the use of grounded theory to capture emergent topics in an inductive manner [33]. A thematic framework was developed once all the domains and topics were created [29]. Using this combination of theory and model, we conducted a deductive analysis based on the different components that can be found in the HBM as well as any other subthemes that were identified in coding, as discussed in the analysis in Matos-Castro et al [15].

The codebook was constructed iteratively, with initial categories that were coded openly. After initial coding, broad categories and emergent subcategories were identified. Subsequent coding used this codebook to code all transcripts. When no additional new codes or themes were identified, the final codebook can be found in Table S1 in Multimedia Appendix 1. Saturation was achieved after no new codes were identified upon examining transcripts. Two members of the research team who had participated in the focus groups did the initial coding to allow insight and perspectives for informing the first stage. Subsequently, to improve reliability and minimize bias, 3 members performed the analysis independently. Intercoder disagreements were discussed, and consensus-based resolutions were applied through team discussions and expanded codebook definitions. Subsequent interpretations were discussed with CTW and CSAC members to ensure adequate and contextual understanding. An example of the focus group interviews can be displayed in Table 1.

**Table 1.** Example of the focus group interviews.

Category	Questions guide for group discussion	Participant’s responses
Knowledge	What comes to mind when you hear the phrase “chronic diseases?”	“It is a condition, which has no particular cure, nor is momentary. From there, it begins to affect patients if they do not care for themselves and, and it becomes a long-term disease.”
Vulnerability	What can you say about chronic diseases present in members of your community or relatives?	“Depression, anxiety, cancer... The progress of chronic conditions is concerning. In our case, in Puerto Rico, it’s alarming to see that previously you would go to offices where people were over sixty, and nowadays, dramatically, from the ages of 20 or 30, you already see patients with chronic diseases*.*”
Barriers	Which difficulties do you consider the community may face regarding the prevention or management of chronic diseases?	“There are people who don’t have health insurance because of what they earn they don’t qualify for the government plan, they get sick, they have to go to appointments and see the doctor, but they don’t do it because they don’t have the money to take care of themselves or to have medical insurance.”

### Ethical Considerations

#### Ethical Approval

This study was approved by the Ponce Health Sciences University Institutional Review Board (protocol number: 1904011672R002, approval date April 14, 2021; last approval August 25, 2023). In response to the COVID-19 pandemic, an addendum was approved to move the protocol to a virtual format, ensuring research continuity while upholding participant privacy and data security standards.

#### Informed Consent

All participants provided informed consent prior to joining the study. The research team explained the study’s objectives, the voluntary nature of participation, possible risks, and the confidentiality measures.

#### Privacy and Confidentiality

Zoom was selected as the videoconferencing platform for its compliance with privacy standards, including encrypted communications, secure (password-protected) sessions, and tools for moderating discussions and providing private support as needed. Only preregistered participants were given access, and all recordings were stored securely and accessible exclusively to the research team. Identifiable information (contact details and names) was handled strictly by the research team. During focus group discussions, participants were assigned numbers, which they used instead of names to maintain anonymity and confidentiality. All personally identifiable information was removed during data analysis and reporting.

#### Compensation

Participants were compensated for their time and contributions in accordance with institutional policies. The compensation provided was US $25, following the completion of each focus group session, at a later date. The amount was set to reimburse any costs that the participant might have had to participate, such as internet use and time.

## Results

### Participant Characteristics

Ten online focus groups were created and were composed of adults aged 21 years or older (N=73) who were residents of the southern regions of Puerto Rico. Of the participants, 77% (n=56) were cisgender women, and 23% (n=17) were cisgender men, with an average age of 46 (SD 14.46) years (refer to Table 2), showing a skewed representation for women. This result was expected, as past experiences and literature suggest that males are reluctant to participate in health research, which might explain this trend [34]. All focus groups were held between 2020 and 2021. Participants were given a stipend for their time and any costs incurred in the participation of focus groups. To obtain this stipend, participants were required to sign a receipt for compensation, which was completed after the focus group sessions at a location agreed upon by both the participant and the research team. This would follow all IRB and HIPAA (Health Insurance Portability and Accountability Act) compliance requirements, with only research team members handling identifiable information, ensuring that all information was maintained anonymously and securely.

**Table 2.** Sociodemographic data.

Variable and characteristic		Participants, n (%)
Gender		
Cisgender Man		17 (23)
Cisgender Woman		56 (77)
Marital status		
Widower		1 (1)
Single/Never Married		22 (30)
Married		37 (51)
Divorced/Separated		10 (14)
Living with a partner		3 (4)
Highest level of education		
High school or less		15 (21)
Technical degree		5 (7)
Associate degree		8 (11)
Bachelor’s degree		27 (37)
Master’s degree		15 (21)
Doctorate’s degree		3 (4)
City of residence		
Peñuelas		7 (9)
Guanica		14 (19)
Guayanilla		16 (22)
Coamo		16 (22)
Santa Isabel		8 (11)
Juana Diaz		12 (16)

### Recruitment, Participation, and Attendance

A participation rate of 86% was achieved in our virtual focus groups, compared to a rate of 55% in previous in-person groups. Minimal dropouts occurred. Various recruitment strategies (community networks, social media, flyers, and CTW/CSAC) contributed to achieving a broad range of health status experiences in the sample.

### Technology, Privacy, and Logistical Outcomes

Possible challenges were identified and addressed through different methods, which are detailed in Table 3.

**Table 3.** Identified challenges and proposed solutions.

Identified challenges	Proposed solutions
Lack of knowledge on the part of the participants about the use of technology.	Research assistants train participants by telephone on the use of technological equipment for the virtual focus group (cell phones, tablets, and computers). This orientation takes place several days before the virtual focus group is held.
Reduce dropout rates	Research assistants will follow up through bidirectional communication with participants on previous days to minimize difficulties that they might encounter leading up to a dropout. For example, assistants called participants days before to clarify doubts and remind them of the date and time of the virtual focus groups.
Participants were in locations with various physical interruptions and privacy concerns.	Research assistants guide participants on the importance of being in a private and comfortable place free of distractions in which they can express themselves freely during the virtual focus group (for example, a room at home).
Participants do not have knowledge about the use of the Zoom app.	Research assistants contacted participants via telephone days before the virtual focus groups with the aim of guiding the participants, step by step, on the use of Zoom and technical support if needed. Test exercises are carried out several days before the virtual focus group is held.
The internet connection goes down, and the participant involuntarily disconnects from the virtual focus group.	The research assistant assigned to that participant quickly contacts the participant by phone to identify and resolve potential technical glitches. If the situation is not resolved, the research assistant becomes the interlocutor, "immediately conveying the participant’s expressions and comments on the discussion topic to the other members of the virtual focus group."
The participant’s internet signal is weak and causes the image to freeze or the audio to not be heard clearly during the virtual focus group.	The research assistant assigned to that participant communicates quickly via telephone and offers them the possibility of continuing their participation in the focus group via telephone or to withdraw from the study, while consistently emphasizing that participation in the group is entirely voluntary.
In-person delivery of the stipend, which required participant signature for the institutional documents.	Research team communicated with participants to agree on a safe location following any health guidelines implemented (eg, open spaces), on a date that worked for the participant. In the case that the participant was not able to arrive at a location, an identified person could serve as a proxy if the participant permitted and provided a written agreement. Following all IRB^a^ and HIPAA^b^ compliance, any identifiable information was handled by the research team and receipts were secured.
Privacy challenges	During meetings with the research team, participants were told in an individual manner about the importance of being in a private and comfortable environment. Zoom links and passwords were only sent on the day of the focus group, making sure that these were sent to the correct email addresses or phone numbers (depending on the preference of the participant). Zoom rooms were password-protected.

a IRB: Institutional Review Board.

b HIPAA: Health Insurance Portability and Accountability Act.

### Focus Group Dynamics and Observations

Participants were told and advised about the importance of being in a private and comfortable environment. Participants were assigned numbers before entering the chat and were recommended to use these numbers instead of names to ensure anonymity, as guided by past groups and behaviors. In practice, participants in the virtual focus groups rarely used these, often using natural conversational references such as “I agree with what she said.” The research team ensured that privacy guidelines were sufficiently explained before and during the sessions to prevent identity disclosure. If names were said, these were censored and replaced with the participant’s number in transcriptions, though this rarely occurred.

Sessions began with moderator-led “ice-breaker” activities to promote open sharing and participation. Research assistants provided technical and emotional support throughout. No incidents of mental health crises required intervention, but all personnel were prepared, and protocols had been established.

### Thematic Analysis Outputs

Key domains and emergent topics were identified through the HBM framework, as detailed in Table 1.

## Discussion

### Principal Findings

After the COVID-19 pandemic began, owing to different health restrictions, many researchers had to find ways to continue their studies without compromising participants’ health. This has led to many studies using videoconferencing software to continue reporting their findings [7,9,35]. As a protocol, our goal is not only to present our findings but also to describe a method that can be built upon and refined in other settings. Several studies discuss how videoconferencing software (in most cases referring to Zoom, the most widely used and available platform for research/educational institutions) allows the continuation of research activities, specifically focus groups. In a study by Halliday et al [7], they emphasized how virtual focus groups reduce logistical barriers, such as travel and costs of hosting physical places while maintaining data quality. Our protocol builds upon these principles and addresses other factors, such as digital exclusion through participant technical support, ensuring balanced access. This emerging field merits investigation, as it could have various advantages that could improve the implementation of qualitative research and, specifically, the focus group experience.

Innovative methods are needed, as qualitative research methods can result in risk factors for transmitting health problems during crises, such as epidemics or pandemics. Thus, creating an environment that allows study individuals to participate without being directly affected by the global COVID-19 pandemic was essential. Using our adaptations can help to promote the participation of groups that experience health differences while assuring the quality of the experience for participants and the integrity of the data collected. However, it is important to acknowledge that this may not be suitable for all research questions or populations. In some cases, such as those that require direct observation of participants, an in-person or hybrid method may be more appropriate. Nevertheless, in contexts that do merit virtual environments, we aim to address the various challenges that are present and demonstrate the virtual method’s advantages.

In recent qualitative focus group studies, selection and social desirability biases were identified because they only attracted participants interested in a specific topic in their interviews [36]. Kloppe et al [37] stated that to overcome this challenge, one must ensure that additional stakeholders are included so they can provide a different perspective; thus, our work with the CTW and CSAC members in identifying communities and recruiting participants helped reduce the likelihood of researchers’ selective sampling based on implicit preferences. Beyond sampling, other studies have identified that these types of interviews and in-person focus groups can bias participants’ responses due to the researcher’s presence [2]. Hence, we understand that it is essential for the moderator to set an environment for open discussion without creating bias owing to their own beliefs and preconceptions.

We implemented various practices to help mitigate these challenges. Sections of the protocol were segmented in a way that research members would only be involved in specific parts and minimize potential bias. We minimized overlaps in tasks. For example, transcription and analysis were done so minimally by members who were directly in the focus groups for initial coding, with a completely different team working independently for coding. Finally, moderators and cofacilitators were to follow question guides developed in conjunction with the community members, making changes in areas to be more culturally relevant to participants, which researchers might not have considered, recognizing that due to inherent subjectivity in qualitative work, it cannot be wholly eliminated but can be diminished.

Creating focus groups that effectively engage hard-to-reach populations presents many challenges. Some cited in the literature include members who are geographically far from where the focus group is being held, the lack of transportation, or participants who have physical health limitations, such as chronic conditions or limited mobility. The latter can be due to different factors, such as individuals who are in active treatment for a condition, are ill, or have been recommended bed rest [17-20]. Excluding such individuals can result in the loss of novel and unique data. By implementing our virtual focus group protocol, we obtained valuable perspectives from these hard-to-reach individuals living with physical health conditions that impede mobility or have geographical restrictions. We believe this to be one of the most important benefits of the virtual focus group modalities. This inclusion should be taken into consideration for future focus group discussions. The voices of these participants are generally not present in a focus group setting; however, this modality allows them to provide data on certain phenomena. Unlike one-to-one interviews, they have the opportunity to interact with other participants.

One participant best stated this:

*Thank you for meeting with us in this manner. I am a cancer patient and can’t get out of bed… In this way, I can participate*.

This approach was initially used as an alternative to in-person focus group sessions due to the COVID-19 restrictions in place; thus, for many researchers, this was their first time using this modality, including ourselves. As a result, the data collected during this period, in which participants were living in a different context due to the pandemic, could have influenced group dynamics. Some studies have reported that virtual focus groups do not produce the desired outcomes and have identified specific issues, such as those discussed by Aligato et al [35] and Lobe et al [38]. Issues identified include the need for notetakers to be more involved in their responsibilities, scheduling problems, higher dropout rates, a distraction from the environment, early departure, technical problems, and privacy concerns.

Maintaining group dynamics in virtual settings can also be difficult due to limits in nonverbal cues and interruptions caused by technical problems. Our use of a cofacilitator and research assistants mitigated these challenges by ensuring active participation and addressing problems in real-time without interrupting the focus group session, thus relieving the moderator of technical problem-solving and reducing session disruptions. In the literature, the group size for virtual focus groups was 4‐6 participants. In our experience, we were able to have group discussions with 8‐10 participants, thanks to the more involved team and through the moderator’s role of facilitating participation, maintaining focus on maintaining discussions aligned with the question guide, and providing an inclusive environment for better rapport. Technical challenges were handled solely by the cofacilitator and research assistants, not intervening during discussions, while the moderator focused fully on focus group discussions as a traditional moderator.

Other issues in this area include not everyone having access to the internet or technology and some lacking the necessary skills [39]. This study design necessitates internet access; a weak internet connection can lead to audio or vocal distortions, dropped calls, and pauses, resulting in unintelligible interview segments [9]. Furthermore, a restricted connection may cause limited data collection when a participant uses only audio and deactivates the camera. This could lead to digital exclusion, where participants are excluded due to not being technologically literate [40]. Though we tried to mitigate this potential problem via in-depth technical help, we cannot discard this possibility.

Additionally, we must consider the ethical implications beyond the ethical protocols implemented for this study, as this research poses further implications that need to be considered when using virtual tools. First, what other measures can researchers implement to better ensure the anonymity of those participating in these groups? Second, to create honest data, what can be done to facilitate a comfortable setting for virtual focus group participants, where safety and well-being can be ensured?

In our experience, we were able to maintain ethical approaches and participant privacy by using internet-based consent forms and ensuring direct communication with researchers to explain these consent forms. During sessions, persons were given numbers, which were used during focus groups so as to protect anonymity. Additionally, participants were encouraged to be in a private and comfortable environment to make sure that all conversations were as private as possible. However, it should be noted that the location was ultimately the participant’s choice, which means that guidance given by the research team has to be comprehensive and should ensure that the participant understands these ethical principles for their own protection.

Even with these identified challenges, we successfully adapted the focus group modality for virtual data collection using videoconferencing software. We implemented several novel aspects that facilitated the process, ensuring a high participation rate and effective data collection. Unlike previous studies that relied solely on participants’ existing resources and knowledge (eg, Nobrega et al [8]), our protocol included a comprehensive orientation session, minimizing the risk of nonparticipation due to lack of technological knowledge. Additionally, studies by Lobe et al [38] emphasized the need for virtual focus groups to have a more structured approach due to different factors. Our approach extended this by incorporating cofacilitators and research assistants who provided real-time technical support and helped maintain group engagement. Another recommendation by this same author is conducting presession technical checks, which our protocol was able to apply before and during participation.

After participants agreed to participate, our research team evaluated their means of communication. This included assessing whether the potential participant had access to devices, such as computers, tablets, or smartphones. Participants also consulted with research assistants to gauge their familiarity with Zoom software. The research team provided step-by-step guidance for those unfamiliar, ensuring they understood how to use the platform. This individualized communication was key to ensuring participant engagement and reducing dropouts.

We also emphasized the role of a cofacilitator, whose responsibilities included handling technical details, scheduling meetings, managing participant screening to ensure only scheduled individuals joined, and serving as a backup moderator in case of technical difficulties. Cofacilitators were trained research team members who were proficient with the software and equipped to manage unforeseen circumstances.

Research assistants played a more involved role in this adaptation. In addition to taking notes and observing focus groups, they maintained direct communication with participants. If a participant disconnected for any reason, their assigned research assistant contacted them via phone or messaging (based on participant preference) to resolve the issue and guide them back to the session. Research assistants also facilitated participation by compiling and sharing text chat messages, ensuring contributions from soft-spoken individuals were included in the discussion, thereby enriching the data.

Prior to implementing the virtual focus groups, participation rates in our in-person sessions were 55% (40/73 participants). We identified several barriers that might have led to these dropouts, such as geographic distance, scheduling problems, and availability of venues that were convenient for all participants. When the COVID-19 pandemic required a shift to virtual modalities, we used these experiences to ensure better participation. We introduced flexible scheduling by offering multiple time blocks for participant preference. Additionally, this modality removed the geographic limitations, as participants could participate using their devices in areas they termed appropriate.

The inclusion of a more involved process for participant participation, such as technical assistance, orientations for the focus groups, and follow-up after participants expressed interest in participation, always emphasizing the voluntary nature of participation and the option to withdraw at any time, encourages more follow-through, this leading to an increase in the participation rate of 86% (63/73) in the virtual modality.

Financial costs appeared to remain minimal (although no itemization of costs was made at the time of implementation). We believe this is likely due to the widespread accessibility of videoconferencing software. These findings demonstrate that, when carefully implemented, virtual focus groups can overcome challenges and provide a valuable platform for qualitative data collection.

This methodology was effective in our case for health differences research due to its ability to facilitate diverse participation, reduce logistical barriers, and provide comfort for participants. However, the protocol could also be adapted for use in other fields, such as education or social work, by adjusting the data collection instruments and tailoring the technical support to meet the specific needs of those fields.

In Nobrega et al [8], virtual focus groups were used to evaluate the impact of a diversity program. The study highlighted several advantages, including a larger geographical reach, easier access for individuals with limited mobility, and greater comfort for participants. In another example, Turner et al [41] used online focus groups for the evaluation of energy use and investment decisions by personality traits. Participants were shown a marketing video and later convened in a virtual focus group setting. In this case, the virtual manner was found to have limitations in regard to participation, but benefits regarding more comfort in participation due to perceived anonymity and more engagement due to the ease of attendance and less worrying about tasks that could be done while conversing. Finally, we can also mention the use of this modality in a study by Borti et al [42], in which virtual focus groups and communications were used to study teachers’ opportunities and knowledge in sub-Saharan Africa using the Ubuntu research paradigm. This used different virtual platforms to establish productive collaborations using virtual groups. Of importance was the need for researchers to support collaborators in accessing the necessary technologies and to take into consideration the platforms that are used.

These examples show that virtual focus group methodologies can be applied in different contexts, such as project evaluations, commercial applications, and social science applications. Key benefits include their ability to overcome geographical barriers, enhance accessibility for diverse participants, and create a comfortable environment conducive to open and honest discussion. These various studies showed that the method was effective for these research goals. With this in mind, we wish to be able to provide a guide that other researchers can use to establish their own virtual focus group, including key elements, such as establishing the necessary members in a research team, addressing technological barriers for participants, and implementing other aspects for a smooth operation. The changes, benefits, and challenges are detailed in Table 4. As seen from these papers, different strategies were applied, but these lacked some of our suggested additions and practices, such as that of comoderators, research assistants, and procedures to facilitate participation. We hope to be able to facilitate these processes with our guide.

**Table 4.** Comparison of traditional focus groups and virtual focus groups, with benefits and possible challenges.

Traditional focus groups	Adaptations in virtual focus groups	Benefits and challenges from adaptation
Informed consent can be read at the site or venue and signed on paper	Informed consent should be read to participants before the session, which could be done through phone or private virtual sessions.	Requires more preparation, but ensures that participants are informed correctly.
A venue is chosen beforehand, and time is established based on participants’ input. It could prove difficult for some members to arrive due to geographical constraints.	Due to the virtual nature of the study, participants will be in their home or most comfortable environment. Participants are advised on best practices to ensure a private environment. Time can be adjusted based on members’ availability. Members might arrive due to technical difficulties.	Requires technical support and follow-up, but leads to better accessibility for participants via the reduction of travel barriers
Led normally by 1 facilitator or moderator who runs the focus group and moderates interactions in a way that there is balanced participation, controlling for dominant participants and encouraging participation through dialogue and nonverbal cues.	Led by a moderator for running the group and a cofacilitator for backup, technical assistance, emotional support if needed, and ensuring participation. Moderation is done through dialogue, but text can also be read, though these should only be used to enrich conversations and dialogue. Fewer nonverbal cues are possible due to limited body vision through webcams.	Team-based moderation is required, with cofacilitator and research assistants taking active roles in real-time monitoring for technical problems. This requires additional training and preparation for the use of virtual conference software but ensures technical issues are minimal to the sessions, which allows moderators to serve their traditional role. Nonverbal cues are kept to a minimum or eliminated if participants opt not to turn on the camera.
Notetakers have a passive role and oversee making important notes of certain interactions, body language, and important topics and writing them down. Usually, 1 or 2 per session	Notetakers, or research assistants in this case, take on a more active role, communicating with participants before and during the sessions to ensure optimal conditions for participation. Serve as support for technical difficulties Facilitator for communication to assigned participants Serve as emotional support if needed. In this case, more than 2 are recommended to cover all participants.	Requires more personnel but ensures participation, follow-ups on losses due to technical issues, and gives a chance for less talkative individuals to communicate via private messages.
Focus group size is generally 6-12 participants.	Focus group size can be done with larger groups but should be limited to 6-8 to ensure better control. More than 10 participants are not recommended.	Smaller groups can facilitate richer and more in-depth discussion. Additionally, with smaller groups, the technical burden is reduced per session. Depending on software use, there might be a limit to the number of screens visible to the moderator; thus, a smaller group is preferred in this instance.

### Limitations and Recommendations for Future Protocols

Although our sampling approach worked for the purposes of our exploratory study, we acknowledge that in different research contexts, greater comparability and homogeneity might be desired. In these cases, we recommend the addition of a prescreening assessment step added to the protocol, which can be done through the use of survey software to ensure that participants comply with inclusion criteria according to the studies’ aims.

Despite the variety of recruitment strategies used (CTW liaisons, printed materials following COVID-19 safety measures, word of mouth, and online outreach), women had a much higher rate of participation (77%). This approach led to a larger reach but did not overcome the gender imbalance, which may also reflect contextual factors and recruitment modalities. Future implementation should consider exploring additional outreach measures that target males, such as using men’s groups, work sites, and organizations. Acknowledging that this gender participation trend is reflective of local realities in community engagement in Puerto Rico, it might limit the transferability of our findings in other contexts.

In Puerto Rico, where the study was conducted, internet penetration is approximately 87.3% [24]. Furthermore, the context of the COVID-19 pandemic led many individuals to acquire digital skills and familiarity with virtual platforms rapidly, facilitating broad participation in virtual focus groups. During recruitment, all participants were confirmed to have the necessary technology. However, it should be noted that this circumstance may not apply in other regions. In settings with lower internet penetration or limited access to technology, researchers may face significant barriers to participation and should consider this a critical limitation when adapting similar protocols.

Recommendations for a quiet and private environment represent an ideal situation rather than an expected result. While our protocol emphasized the importance of contacting participants before focus groups to provide instruction and offer advice on best practices, this does not guarantee that participants will follow them. Ultimately, this is a decision that lies with the participant; thus, they should not be pressured or expected to have cameras on. This can be considered an inherent limitation to this methodology. Though in our experience with proactive support and attending to the participants’ technical needs before the group, very few elected not to show their camera.

Although the research team managed stipend delivery during the COVID-19 emergency, future studies, especially those covering larger geographic areas, may benefit from empowering trained community members to assist with this process. Leveraging community-led teams for stipend distribution could increase efficiency, reduce logistical burden for research staff, and foster deeper community partnership, supporting both scalability and sustainability of qualitative research protocols. Additionally, one could implement electronic signatures and use electronic payment measures to ensure the least amount of contact occurs between the research team and participants.

Several safeguards were established to protect participant privacy. The nature of audio and video recording presents an inherent privacy risk that cannot be fully eliminated. While measures, such as assigning numbers and changing display names, can manage some aspects of anonymity, each participant’s face and voice are still tied to their contribution. Thus, transparency must be exercised by researchers, and they must adopt comprehensive privacy protocols as described, while also exploring additional methods that could enhance anonymity in future virtual focus groups.

It should be noted that although participation increased in virtual groups (86%) compared to in-person groups (55%), this comparison occurred in distinct time periods and under changing circumstances, such as the COVID-19 pandemic, which might have given participants more time to participate. Additionally, other factors, such as community outreach, flexible schedules, internet penetration of approximately 87.3% [24], and technical support, may have contributed to these results. These circumstances can vary by location, and in cases where there is lower internet penetration or limited access to technology, researchers may face significant barriers. This represents a limitation that should be considered when adapting this protocol in different areas.

Thus, implementations should be taken cautiously. However, we still believe that if planned according to the participants’ time and increased accessibility, this could lead to high participation. Similarly, we perceived the costs for virtual focus groups as minimal due to savings in venues and travel, but no formal itemized cost analysis was carried out. Thus, future studies might wish to use a prospective approach to compare direct costs.

### Conclusion

We believe that virtual focus groups are an important and effective tool to facilitate the participation of undersupported populations, especially those who, for health reasons or lack of transportation, find it difficult or cannot reach a physical place to participate in traditional focus groups. Likewise, our virtual focus group protocol can be used anytime and be applied in different scenarios. It is an effective tool for continuing qualitative research and collecting information in real-time and reducing barriers that could impede the process. Following our protocol, past challenges were mitigated, we found an increased participation rate, and an adapted modality was established for future qualitative research.

## Supplementary material

10.2196/77937 Multimedia Appendix 1 Codebook of themes and quotations.

## Abbreviations

ATSDR: Agency for Toxic Substances and Disease Registry
CBPR: Community-Based Participatory Research
CDC: Centers for Disease Control and Prevention
CEC: Community Engagement Core
CSAC: Community Scientific Advisory Committee
CTW: Community-Trained Workforce
HBM: Health Belief Model
HIPAA: Health Insurance Portability and Accountability Act
IRB: Institutional Review Board
PHSU: Ponce Health Sciences University
RCMI: Research Center for Minority Institutions
SVI: Social Vulnerability Index

## References

[1] Jiang Q, Cohen NL (2020). Use of online focus groups for nutrition and health studies: the Northeast Regional Research Project Experience. Top Clin Nutr.

[2] Creswell JW, Creswell JD (2022). Research Design: Qualitative, Quantitative, and Mixed Methods Aproaches.

[3] Leung FH, Savithiri R (2009). Spotlight on focus groups. Can Fam Physician.

[4] Mishra L (2016). Focus group discussion in qualitative research. TechnoLearn.

[5] Dos Santos Marques IC, Theiss LM, Johnson CY (2021). Implementation of virtual focus groups for qualitative data collection in a global pandemic. Am J Surg.

[6] Rupert DJ, Poehlman JA, Hayes JJ, Ray SE, Moultrie RR (2017). Virtual versus in-person focus groups: comparison of costs, recruitment, and participant logistics. J Med Internet Res.

[7] Halliday M, Mill D, Johnson J, Lee K (2021). Let’s talk virtual! Online focus group facilitation for the modern researcher. Res Social Adm Pharm.

[8] Nobrega S, El Ghaziri M, Giacobbe L, Rice S, Punnett L, Edwards K (2021). Feasibility of virtual focus groups in program impact evaluation. Int J Qual Methods.

[9] Pocock T, Smith M, Wiles J (2021). Recommendations for virtual qualitative health research during a pandemic. Qual Health Res.

[10] Crocker ME, Stout JW (2023). A qualitative study of perspectives on the acceptability and feasibility of “virtual home visits” for asthma. BMC Public Health.

[11] Pugmire J, Lever Taylor J, Wilkes M, Wolfberg A, Zahradka N (2022). Participant experiences of a COVID-19 virtual clinical study using the current health remote monitoring platform: case study and qualitative analysis. JMIR Form Res.

[12] Schlaudecker JD, Goodnow K (2021). The virtual patient and family advisory council in the COVID-19 era. J Am Board Fam Med.

[13] Zhou Z, Li J, Wang H (2023). Experience of using a virtual reality rehabilitation management platform for breast cancer patients: a qualitative study. Support Care Cancer.

[14] Buddle EA, Ankeny RA, Paxton R, Harms RJ, Bray HJ (2024). Emergent design and unanticipated ideas in asynchronous online focus groups: finding an unexpected silver lining in apparent methodological compromise. Int J Qual Methods.

[15] Matos-Castro JC, Ramos-Lucca A, Rosa-Jiménez AA (2023). A qualitative approach to explore perceptions, opinions, and beliefs of communities who experienced health disparities towards chronic health conditions. Int J Environ Res Public Health.

[16] Gosling SD, Mason W (2015). Internet research in psychology. Annu Rev Psychol.

[17] Adler CL, Zarchin YR (2002). The “virtual focus group”: using the Internet to reach pregnant women on home bed rest. J Obstet Gynecol Neonatal Nurs.

[18] Sweet C (2001). Designing and conducting virtual focus groups. Qualitative Mrkt Res: An Int J.

[19] Underhill C, Olmsted MG (2003). An experimental comparison of computer-mediated and face-to-face focus groups. Soc Sci Comput Rev.

[20] Tates K, Zwaanswijk M, Otten R (2009). Online focus groups as a tool to collect data in hard-to-include populations: examples from paediatric oncology. BMC Med Res Methodol.

[21] Wilson I, Daniels N, Gillen P, Casson K (2022). Perspectives on reporting non-verbal interactions from the contemporary research focus group. Nurse Res.

[22] Tudor C (2022). The impact of the COVID-19 pandemic on the global web and video conferencing SaaS market. Electronics (Basel).

[23] Bishop W, Faverio M, Dawson W Internet, broadband fact sheet. Pew Research Center.

[24] ITU (2024). Percentage of population using the internet in Puerto Rico from 2010 to 2022 [Graph].

[25] Xia R, Stone JR, Hoffman JE, Klappa SG (2016). Promoting community health and eliminating health disparities through community-based participatory research free. Phys Ther.

[26] (2024). Social vulnerability index. ATSDR.

[27] Almond N, Deal AM, Page A, Nyrop KA, Muss HB (2025). Associations of social vulnerability index with patient-reported outcomes in women treated with chemotherapy for early-stage breast cancer. Oncologist.

[28] Tong A, Sainsbury P, Craig J (2007). Consolidated Criteria for Reporting Qualitative Research (COREQ): a 32-item checklist for interviews and focus groups. Int J Qual Health Care.

[29] Gibbs G (2007). Analyzing Qualitative Data.

[30] (2021). HIPAA compliance. Zoom.

[31] Katirayi L, Akuno J, Kulukulu B, Masaba R (2021). “When you have a high life, and you like sex, you will be afraid”: a qualitative evaluation of adolescents’ decision to test for HIV in Zambia and Kenya using the health belief model. BMC Public Health.

[32] Mehta P, Sharma M, Lee RC (2013). Using the health belief model in qualitative focus groups to identify HPV vaccine acceptability in college men. Int Q Community Health Educ.

[33] Starks H, Trinidad SB (2007). Choose your method: a comparison of phenomenology, discourse analysis, and grounded theory. Qual Health Res.

[34] Ryan J, Lopian L, Le B (2019). It’s not raining men: a mixed-methods study investigating methods of improving male recruitment to health behaviour research. BMC Public Health.

[35] Aligato MF, Endoma V, Wachinger J (2021). “Unfocused groups”: lessons learnt amid remote focus groups in the Philippines. Fam Med Community Health.

[36] Bergen N, Labonté R (2020). “Everything is perfect, and we have no problems”: detecting and limiting social desirability bias in qualitative research. Qual Health Res.

[37] Kloppe T, Tetzlaff B, Mews C, Zimmermann T, Scherer M (2022). Interprofessional collaboration to support patients with social problems in general practice-a qualitative focus group study. BMC Prim Care.

[38] Lobe B, Morgan DL, Hoffman K (2022). A systematic comparison of in-person and video-based online interviewing. Int J Qual Methods.

[39] Muwanguzi M, Lugobe HM, Ssemwanga E (2021). Retention in HIV care and associated factors among youths aged 15-24 years in rural southwestern Uganda. BMC Public Health.

[40] Wilson-Menzfeld G, Erfani G, Young-Murphy L (2025). Identifying and understanding digital exclusion: a mixed-methods study. Behav Inf Technol.

[41] Turner P, Rushby T, Gauthier S (2021). An online “face to face” focus group approach for understanding how household energy use and green investment decisions differ by personality traits. Int J Qual Methods.

[42] Borti AM, Maurya RK, Jones-Mensah IS, Wickramaarachchi TI (2024). Using the research paradigm of Ubuntu to unpack how Ghanaian novice teachers and their collaborators engaged virtually in collaborative international qualitative researc. Int J Qual Methods.

