# Supplementary material for: Implementation of Virtual Focus Groups as an Effective Strategy in Qualitative Research to Engage With Undersupported Communities: Protocol for Virtual Focus Groups
Source: JMIR Res Protoc. 2025 Dec 8;14:e77937. doi: 10.2196/77937 (PMC12685286; doi:10.2196/77937)
Supplement: Multimedia Appendix 1 [file resprot-v14-e77937-s001.docx]

Supplementary Table 1. Codebook of Themes and Sub-Themes for Analysis

| Themes and Sub-themes | Quotations |
| --- | --- |
| **I. Knowledge** | - |
| **I.A Definition** | 76 |
| **I.A.A Classifications** | 2 |
| **I.A.A.1 Mental health** | 170 |
| **I.A.A.10 Other** | 68 |
| **I.A.A.2 Cardiovascular Health** | 62 |
| **I.A.A.3 Neurological** | 43 |
| **I.A.A.4 Metabolic Disorders** | 118 |
| **I.A.A.5 Rheumatic** | 12 |
| **I.A.A.6 Infectious** | 28 |
| **I.A.A.7 Cancer** | 79 |
| **I.A.A.8 Psychoneuro-immunology** | 17 |
| **I.A.A.9 Respiratory** | 35 |
| **I.B Access to Information** | 51 |
| **I.B.1 Benefits** | 145 |
| **I.B.2 Barriers** | 210 |
| **I.C Prevention Strategies** | 27 |
| **I.C.1 Primary** | 138 |
| **I.C.2 Secondary** | 73 |
| **I.C.3 Tertiary** | 110 |
| **I.D Concerns** | - |
| **I.D.1 Chronic Illness in Childhood/Family** | 65 |
| **I.D.2 Sexually Transmitted Diseases** | 1 |
| **I.D.3 Access to Professional Help** | 137 |
| **I.D.4 Economic Barriers** | 79 |
| **I.D.5 Concern about symptoms and disease progression** | 32 |
| **II. Vulnerability** | - |
| **II.A.1 Individual** | 432 |
| **II.A.2 Familiar** | 139 |
| **II.A.3 Community** | 244 |
| **III. Barriers** | - |
| **III.A.1 Individual** | 195 |
| **III.A.2 Socio-Structural Factors** | 100 |
| **III.A.2.a Economic** | 197 |
| **III.A.2.b Political** | 108 |
| **III.A.2.c Access to Communications and Technology** | 37 |
| **III.A.2.d Access to Services** | 164 |
| **IV Identified Resources** | - |
| **IV.A Support** | 2 |
| **IV.A.1 Individual** | 86 |
| **IV.A.2 Familiar** | 38 |
| **IV.A.3 Community** | 196 |
| **IV.B Recommendations** | 219 |

Supplementary Table 1 presents the codebook for qualitative analysis, listing the main themes and sub-themes identified in focus group discussions, along with the number of quotations assigned to each. The codebook was initially structured around the domains of the Health Belief Model (HBM), with additional themes and subthemes inductively incorporated as they emerged during transcript analysis.
